# Restricted and repetitive behaviors and association with cognition and adaptive functioning in children with autism spectrum disorder in Singapore

**DOI:** 10.3389/fpsyt.2023.1249071

**Published:** 2023-11-16

**Authors:** Wanyun Lin, Yiong Huak Chan, Jennifer S. H. Kiing, Tammy S. H. Lim, Shang Chee Chong, Ying Qi Kang, Ramkumar Aishworiya, Kalyani Vijayakumar Mulay, Mae Yue Tan

**Affiliations:** ^1^Child Development Unit, Khoo Teck Puat-National University Children's Medical Institute, National University Health System, Singapore, Singapore; ^2^Department of Paediatrics, Yong Loo Lin School of Medicine, National University of Singapore, Singapore, Singapore; ^3^Biostatistics Unit, Yong Loo Lin School of Medicine, National University of Singapore, Singapore, Singapore

**Keywords:** restricted and repetitive behaviors, autism spectrum disorder, children, cognition, adaptive functioning, Singapore, Asian

## Abstract

**Background:**

One of the core features of autism spectrum disorder (ASD) is restricted, repetitive patterns of behavior, interests and activities (RRBs). RRBs are known to adversely affect cognition and adaptive functioning. We explored the relationship of RRBs with cognition and adaptive functioning in children with ASD in an Asian setting.

**Methods:**

This cross-sectional study was conducted at a tertiary developmental pediatrics center in Singapore from September 2019 to October 2021. Parent-child dyads (parents and their children ≤7 years old diagnosed with ASD) were recruited. Parents completed the Repetitive Behavior Questionnaire-2 (RBQ-2), which reports total score and two subscales – Motor/Sensory Behaviors (RBQ-2 MS) and Rigidity/Routines/Preoccupation with Restricted Interests (RBQ-2 RRPRI). Standardized assessments included Mullen Scales of Early Learning (MSEL) and Vineland Adaptive Behavior Scales (VABS-II). Data analysis utilized descriptive statistics and Pearson’s correlation.

**Results:**

Parents of 113 children [75.2% male, mean (SD) age 5.0 (1.2) years] participated. Median (IQR) RBQ-2 score was 29.0 (11.0). Significant negative correlations (adjusted for age, gender and family history of ASD) were observed for total RBQ-2 scores with MSEL ELC scores (*r* = −0.248, *n* = 101, *p* = 0.014) and VABS-II ABC scores (*r* = −0.281, *n* = 88, *p* = 0.009). Specifically, these correlations of fair strength were seen only with the RBQ-2 MS subscale for both ELC (*r* = −0.321, *n* = 101, *p* = 0.001) and ABC (*r* = −0.3478, *n* = 88, *p* = 0.001).

**Conclusion:**

In children with ASD, severity of RRBs correlated with adverse cognition and adaptive functioning measures in our study, consistent with Western literature. While our study does not show causality, it adds to literature serving as a foundation for further research for both clinicians and researchers to target RRBs in improving outcomes with children in ASD.

## Introduction

Autism spectrum disorder (ASD) is a neurodevelopmental disorder characterized by deficits in social communication and social interaction, and restricted, repetitive patterns of behavior, interests and activities (RRBs) (1). RRBs describe various behaviors characterized by repetition, inflexibility and inappropriateness. These behaviors frequently lack a clear function and a specific purpose (2, 3). They are deemed pathological when they interfere with social relationships and impede daily activities (4). The Diagnostic and Statistical Manual of Mental Disorders, Fifth Edition (DSM-V) divides RRBs into four subtypes: (a) stereotyped or repetitive motor movements, use of objects, or speech; (b) insistence on sameness, inflexible adherence to routines, or ritualized patterns of verbal or non-verbal behavior; (c) highly restricted, fixated interests that are abnormal in intensity or focus; and (d) hyper or hypo-reactivity to sensory input, or unusual interests in sensory aspects of the environment (1). Compared to social communication impairments, RRBs are less extensively studied in ASD research (4), despite being an independent predictor of ASD prognosis (5).

RRBs are not specific to ASD. They are observed in varied psychiatric and developmental conditions such as Prader-Willi syndrome (6), Fragile X syndrome (7), general intellectual disability and blindness (8). They are also seen in non-autistic children (9). However, early onset of RRBs in children at risk for ASD can predict diagnosis of ASD as well as reduced social engagement (10). RRBs in children with ASD tend to be more excessive and diverse compared to children without ASD (11). It is postulated that RRBs interfere with children’s ability to engage in the external environment where learning opportunities that would promote cognitive and social communication development are present, leading to delayed development of these skills (12).

In children with ASD, RRBs have been associated with impairment in various developmental domains—children with higher levels of RRBs are more likely to present with lower cognition, adaptive functioning, communication, and socialization skills (13–17). Bishop et al. (13) showed associations of RRBs with cognition when assessed using various intellectual quotient measures. Cuccaro et al. (14) and Gabriels et al. (15) showed that lower non-verbal cognitive ability was highly correlated with higher total repetitive behavior scores in children with ASD. A study done by Mooney et al. (16) on children aged 20–55 months showed correlation between higher RRB and lower adaptive behavior composite. Lam et al. (17) in their factor analysis with a population of individuals with ASD from age 20 months to 29   years showed association of RRBs with social and communication impairments.

The relationship between RRBs and cognitive and adaptive functioning has been postulated to have different mechanisms and theoretical frameworks. Theories suggest that RRBs interfere with a person with ASD’s ability to engage in his/her external or social environment where learning opportunities are present (12, 18). Nadig et al. (19) suggests similarly that RRBs reduces likelihood of positive interaction with peers, affecting learning and socialization. Neuropsychological studies have shown that in individuals with RRBs, impairments in behavioral inhibition, cognitive flexibility and monitoring for responses can make integration of a dynamic environment difficult- in turn inciting non-adaptive behaviors (20), which could then potentially affect cognitive and adaptive functioning. A systematic review by Beauchaine (21) also posits that through the neurovisceral integration model, cognitive inflexibility (such as RRBs in ASD) arises from biological inflexibility in the central nervous system- and this itself has been implicated in impaired social skills, impaired executive functioning, and increased behavioral problems, contributing to impaired cognitive and adaptive functioning.

ASD is a neurodevelopmental disorder that manifests differently in individuals from various cultural and ethnic backgrounds (22–25). Matson et al. (22) found significant differences in overall ASD symptom severity and endorsement between multinational groups of Greece, Italy, Japan, Poland, and the United States. Donohue et al. (23) studied parent reports of concerns and found that compared to white parents, black parents reported significantly lesser autism concerns and fewer RRB concerns. Daley et al. (24) highlighted how awareness and concept of autism symptoms including RRBs, vary from culture to culture and advocated the need for culturally informed research. Similarly, Mandell et al. (25) in their review described how culture influences the presentation of ASD and interpretation of its symptoms while informing beliefs about the cause and course of ASD.

So far, no studies have explored RRBs in the Asian pediatric population. We thus aimed to describe RRBs as well as explore the relationship of RRBs with cognitive and adaptive functioning in Asian children with ASD. We also explored how RRBs were related to social behavior. This is the first study to date exploring RRBs in children with ASD in Singapore. We hypothesized that the presence and the severity of RRBs would negatively correlate with cognitive and adaptive functioning, given previous Western studies and what is currently known in the theoretical framework in the relationship of RRBs to cognitive and adaptive functioning. We also investigated whether different types of RRBs are associated with specific profiles of cognitive and adaptive functioning. Potential benefits from our study would be increasing awareness of the prevalence of RRBs in our patients with ASD and its impact on functioning - this would be beneficial for both researchers and clinicians as it will serve as a foundation for further research into whether intervening on RRBs through parental education, behavioral and/or pharmacological interventions could reduce the burden of RRBs and improve clinical outcomes for children with ASD.

## Methods

### Study design and participants

In this cross-sectional study, parent/caregiver-child dyads were recruited over a 2-year period (September 2019 to October 2021) from a tertiary developmental pediatric clinic in Singapore. This clinic is one of two designated public hospital centers in Singapore serving young children with developmental and behavioral conditions. Both institutions function along the same model of care. We support around 13,000 outpatient visits annually, accounting for 14% of our total pediatric outpatient visits in our hospital. This amounts to 35% of the national developmental & behavioral pediatric population served annually in national institutions (26). Participants were recruited if (a) they were aged ≤7 years old and diagnosed with ASD using the Autism Diagnostic Observation Schedule-2 (ADOS-2) (27), and (b) their parents/caregivers were proficient in English. Recruitment occurred during routine clinical visits to the clinic. As part of our routine clinical care for children with ASD, they underwent standardized cognitive and adaptive behavior assessments, which were administered in the clinic. Consenting participants completed standardized questionnaires and consented to the use of data from the clinical assessments. We describe the study measures below. As this was a cross-sectional initial pilot study to explore associations, sample size calculation was not conducted.

### Study measures

We utilized the Repetitive Behavior Questionnaire, Second Edition (RBQ-2) to assess RRBs, and the Social Responsiveness Scale, Second Edition (SRS-2), to measure social ability in different settings (home and school). We also used standardized developmental assessments such as the Mullen Scales of Early Learning (MSEL) and the Vineland Adaptive Behavior Scales (VABS-II) to measure cognitive and adaptive function, respectively. Apart from the RBQ-2 which was administered for study purposes, the other three measures were part of routine clinical care.

The RBQ-2 (9) is a 20-item questionnaire designed for parents to record the frequency and intensity of repetitive behaviors known to occur in both children with and without ASD. Results from the original study showed that the RBQ-2 has good internal consistency and validity, indicating that it is a reliable instrument for measuring a range of repetitive behaviors. Summation of the responses (“scores”) for each of the 20 questions yields a total score (RBQ-2 Total; range: 20–60); a higher total score indicates more frequent and significant RRBs. In this study, we utilized the 2-subscale configuration and report the scores for these—Motor/Sensory Behaviors (RBQ-2 MS), score range: 9–33, and Rigidity/Routines/Preoccupation with Restricted Interests (RBQ-2 RRPRI), score range: 8–24. The RBQ-2 reports frequency and intensity of the RRBs and does not report a dichotomous “yes,” or “no” result. We selected this tool as it is used in children as young as 15 months of age, which was appropriate for use in our clinical setting compared to other tools to measure RRB for example the RBS-R which was for children aged 6 and above (28). While the ADOS-2 results also report RRB scores, we used the ADOS-2 to ascertain inclusion criteria. Our patients in this study also had the Autism Diagnostic Interview-Revised (ADI-R) done with the ADOS-2. While the ADOS-2 and ADI-R do report RRBs in its constructs, we selected the RBQ-2 tool which is more readily clinically available that can be easily and efficiently used in daily practice to measure RRBs, to see how it will map into cognitive and adaptive outcomes.

We administered MSEL to all the participants after the first clinic visit. The MSEL assesses early cognitive and motor development in children (from birth to 68 months) by producing scores representing developmental ages for five subscales—one motor (gross motor) and four cognitive (fine motor, visual reception, expressive language, and receptive language) (29). Scores for each subscale are generated based on a standard mean score of 50 [standard deviation (SD) 10]. The four cognitive subscales’ scores can also be combined to yield an Early Learning Composite (ELC), based on a mean T-score of 100 (SD 15). For young children, the ELC is considered equivalent to a traditional intellectual quotient (IQ) score or a developmental standard score as a measure of overall cognitive function.

The VABS-II is a measure of adaptive function of an individual (from birth to 90 years of age) in five specific domains (communication, daily living skills, socialization, motor skills, and maladaptive behavior) (30). For each domain, the standard mean score is 100 (SD 15); lower scores indicate greater functional impairment. The communication, daily living, and socialization domains make up the Adaptive Behavior Composite (ABC), a standardized score of the overall level of adaptive function, which directly maps to core ASD symptoms and impact on daily function. The VABS-II assessment was performed by trained psychologists.

The SRS-2 distinguishes ASD from other conditions by identifying the presence and severity of social impairment in individuals with ASD, and measures deficits in social behavior across 5 subscales (social awareness, social cognition, social communication, social motivation, and restricted interests and repetitive behavior) (31). The overall total score and individual subscale scores are reported as T-scores [mean (SD) of 50 (10)]. It has 4 rating forms, of which two of them (the Preschool form, for ages 2.5–4 years, and the School-Age form, for ages 4–18 years) are intended to be completed by the parent or teacher of the individual with ASD. In our study, parents and/or parent-appointed teachers completed either of these forms (depending on the age of the child) after the child’s first clinic visit. If the teacher SRS-2 score was not available, only the parent SRS-2 score was recorded.

### Ethics approval

Institutional review board approval was obtained prior to the commencement of the study (National Healthcare Group Domain Specific Review Board [NHG DSRB], Reference Number: 2019/00132). Consent was obtained from either parents or caregivers of the children with ASD.

### Statistical analyses

All analyses were performed using IBM SPSS Statistics for Windows, Version 28.0 (Armonk, NY: IBM Corp). Statistical significance was set at *p* < 0.05 (2-sided). Internal consistency of the RBQ-2 was assessed using Cronbach’s alpha (α). Descriptive statistics are presented as mean (SD) for normally distributed numerical data; median (IQR) for numerical results on a non-normal distribution; and, n (%) for categorical variables. Strength of associations was assessed using Pearson’s correlation. Pearson’s correlation coefficient of 0.3–0.59 describes a fair correlation, and <0.3 describes a weak correlation (32).

## Results

### Participant characteristics

The parents of 113 children participated in this study; the children’s demographics are shown in Table 1. A positive family history of ASD was present in 12.4%. A third of the parents (either mother or father) had a graduate degree and above. A total of 70.8% received financial assistance for medical care.

**Table 1:** Demographics of study participants (*n* = 113), with median scores for scores for RBQ-2, MSEL, VABS-II, and SRS-2 for study participants.

Characteristic	Children with ASD (*n* = 113)
Age, years (mean, SD)	5.0 (1.2)
Male, *n* (%)	85 (75.2)
Positive family history of ASD, *n* (%)	14 (12.4)
**Level of paternal education, *n* (%)**	
Primary/secondary	14 (12.4)
Post-secondary/diploma	29 (25.7)
Graduate degree and above	34 (30.1)
Not known	36 (31.9)
**Level of maternal education, *n* (%)**	
Primary/secondary	15 (13.3)
Post-secondary/diploma	22 (19.5)
Graduate degree and above	39 (34.5)
Not known	37 (32.7)
Received financial assistance, *n* (%)	80 (70.8)
**Study measure**	**Score, median (IQR)**
RBQ-2 total	29.0 (11.0)
RBQ-2 MS	12.0(5.0)
RBQ-2 RRPRI	11.0 (3.0)
MSEL ELC [*n* = 12 (10.6%) missing]	60.0 (32.0)
VABS-II ABC [*n* = 25 (22.1%) missing]	73.0 (11.0)
Parent-reported SRS-2 [*n* = 35 (31.0%) missing]	62.5 (12.0)
Teacher-reported SRS-2 2 [*n* = 51 (45.1%) missing]	76.0 (13.0)

ABC, Adaptive Behavior Composite; ASD, Autism Spectrum Disorder; ELC, Early Learning Composite; IQR, Interquartile Range; MSEL, Mullen Scales of Early Learning; RBQ-2, Repetitive Behavior Questionnaire, Second Edition; RBQ-MS, RBQ Motor/Sensory Behaviors; RBQ-RRPRI, RBQ Rigidity/Routines/Preoccupation with Restricted Interests; SRS-2, Social Responsiveness Scale, Second Edition; VABS-II, Vineland Adaptive Behavior Scales, Second Edition.

Scores for the study measures are presented in Table 1. Cronbach’s α for the RBQ-2 was 0.852. Median RBQ-2 Total was 29.0 (IQR 11.0). The MSEL ELC had a median of 60.0 (IQR 32.0) which was in the ‘very low to below average’ range, while the VABS-II ABC had a median of 73.0 (IQR 11.0); this was in the ‘moderately low’ range. No significant correlations were observed between RBQ-2 Total and participant characteristics (age, gender, positive family history of ASD, financial status, parent’s education level).

### Relationship between RRBs and cognitive functioning

Table 2 reports the correlations between the RBQ-2 and the MSEL. We adjusted for confounders of gender, age, and a positive family history of autism spectrum disorder. The RBQ-2 Total was negatively correlated with weak strength with the MSEL ELC (*r* = −0.248, *p* = 0.014). Specifically, this correlation was seen only with the RBQ-2 MS subscale, with fair strength here (*r* = −0.321, *p* = 0.001).

**Table 2:** Association between RBQ-2 and MSEL scores.

		MSEL									
		Visual perception		Fine motor		Receptive language		Expressive language		ELC	
		*r*	*r*^	*r*	*r*^	*r*	*r*^	*r*	*r*^	*r*	*r*^
RBQ-2	RBQ-2 total	−0.316*	−0.304*	−0.126	−0.121	−0.284*	−0.260*	−0.215*	−0.215*	−0.265*	−0.248*
	RBQ-2 MS	−0.349**	−0.338*	−0.242*	−0.248*	−0.333*	−0.306*	−0.254*	−0.248*	−0.334*	−0.321*
	RBQ-2 RRPRI	−0.207	−0.181	0.001	0.037	−0.165	−0.142	−0.112	−0.099	−0.139	−0.108

ELC, Early Learning Composite; MSEL, Mullen Scales of Early Learning; RBQ-2, Repetitive Behavior Questionnaire, Second Edition; RBQ-MS, RBQ Motor/Sensory Behaviors; RBQ-RRPRI, RBQ Rigidity/Routines/Preoccupation with Restricted Interests. *r*^, adjusted *r* for gender, age, and positive family history of autism spectrum disorder. *N* = 101 for *r* & *N* = 96 for *r*^. *p < 0.05, **p < 0.001. Bolded values are statistically significant results.

**Figure 1:**
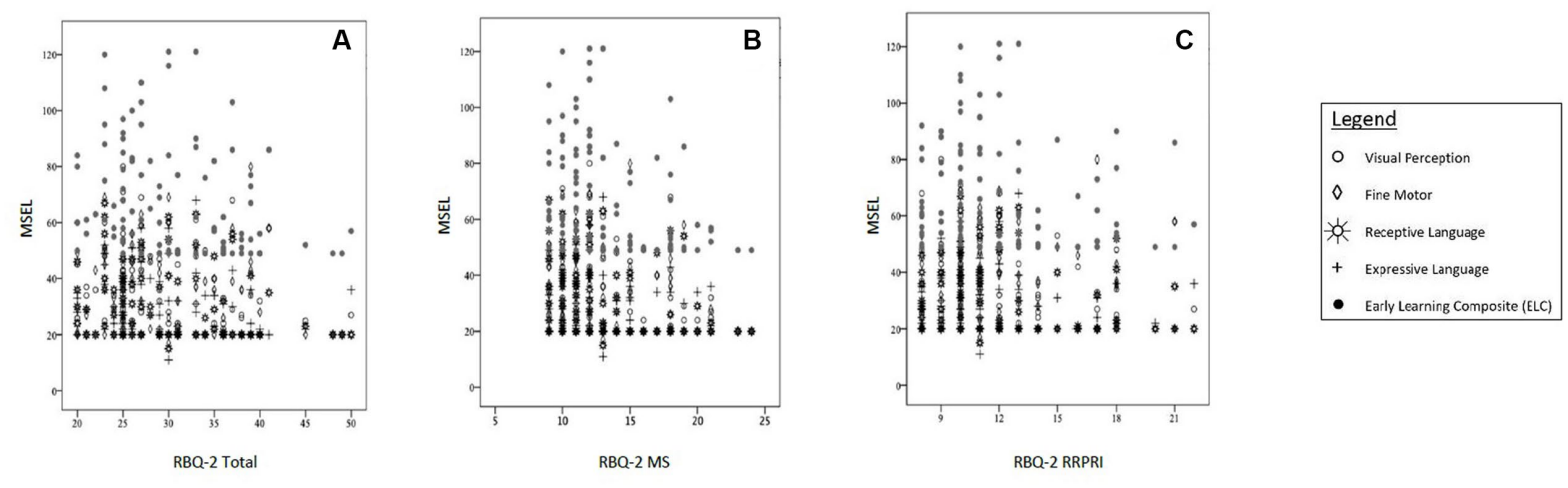
Correlations between MSEL scores [subscales (Visual Perception, Fine Motor, Receptive Language, Expressive Language) and Early Learning Composite (ELC)] and **(A)** RBQ-2 Total score, **(B)** RBQ-MS score; and **(C)** RBQ-2 RRPRI score. MSEL, Mullen Scales of Early Learning; RBQ-2, Repetitive Behavior Questionnaire-2; RBQ-2 Total, RBQ-2 Total score; RBQ-2 MS, RBQ-2 Motor/Sensory Behaviors subscale score; RBQ-2 RRPRI, RBQ-2 Rigidity/Routines/Preoccupation with Restricted Interests subscale score.

Further analysis revealed that significant negative correlations existed between RBQ-2 Total and 3 MSEL subscales (visual perception, receptive and expressive language). Higher RBQ-2 MS scores were significantly associated with lower fine motor scores on the MSEL; however, a similar correlation did not exist between the latter and RBQ-2 Total score. Looking at the specific domains of the MSEL, the visual perception subscale score had the largest negative correlation (fair strength) with both RBQ-2 total and RBQ-MS scores. These results are represented graphically in Figure 1.

### Relationship between RRBs and adaptive functioning

Table 3 reports the correlations between the RBQ-2 and the VABS-II. Similarly, we adjusted for confounders of gender, age, and a positive family history of autism spectrum disorder. The RBQ-2 Total was negatively correlated with the VABS-II ABC, with weak strength (*r* = −0.281, *p* = 0.009). Similar to the MSEL ELC, this correlation was seen only with the RBQ-2 MS subscale for the ABC (*r* = −0.347, *p* = 0.001, showing a fair correlation). Looking at the specific domains of the VABS-II, the communication subscale score had the largest negative correlation with both RBQ-MS and RBQ-RRPRI scores.

**Table 3:** Association between RBQ-2 and VABS-II scores.

		VABS-II									
		Communication skills		Daily Living skills		Socialization skills		Motor skills		ABC	
		*r*	*r*^	*r*	*r*^	*r*	*r*^	*r*	*r*^	*r*	*r*^
RBQ-2	RBQ-2 total	−0.321*	−0.321*	−0.232*	−0.214*	−0.207	−0.228*	−0.184	−0.213	−0.284*	−0.281*
	RBQ-2 MS	−0.370*	−0.369*	−0.283*	−0.258*	−0.262*	−0.294*	−0.285	−0.326	−0.348*	−0.347*
	RBQ-2 RRPRI	−0.223*	−0.218*	−0.137	−0.147	−0.130	−0.136	−0.020	−0.042	−0.175	−0.177

ABC, Adaptive Behavior Composite; RBQ-2, Repetitive Behavior Questionnaire, Second Edition; RBQ-MS, RBQ Motor/Sensory Behaviors; RBQ-RRPRI, RBQ Rigidity/Routines/Preoccupation with Restricted Interests; VABS-II, Vineland Adaptive Behavior Scales, Second Edition. *r*^, adjusted *r* for gender, age, and positive family history of autism spectrum disorder. *N* = 88 for *r* & *N* = 83 for *r*^. *p < 0.05, **p < 0.001. Bolded values are statistically significant results.

**Figure 2:**
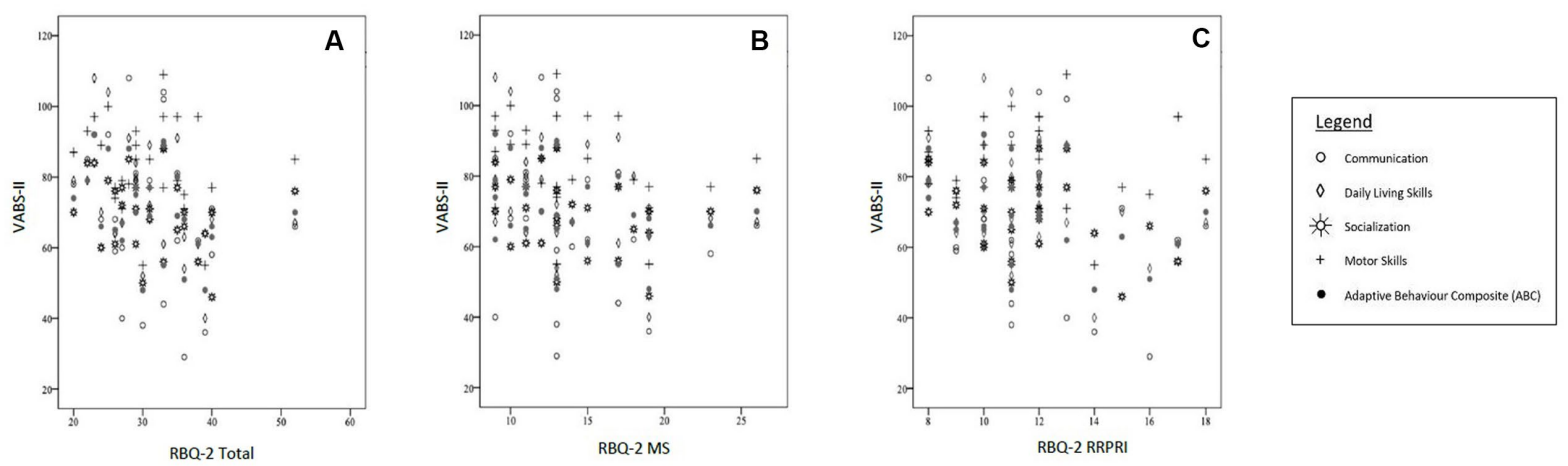
Correlations between VABS-II scores [subscales (Communication, Daily Living Skills, Socialization, Motor Skills) and Adaptive Behavior Composite (ABC)] and **(A)** RBQ-2 Total score, **(B)** RBQ-MS score; and **(C)** RBQ-2 RRPRI score. RBQ-2, Repetitive Behavior Questionnaire-2; RBQ-2 Total, RBQ-2 Total score; RBQ-2 MS, RBQ-2 Motor/Sensory Behaviors subscale score; RBQ-2 RRPRI, RBQ-2 Rigidity/Routines/Preoccupation with Restricted Interests subscale score; VABS-II, Vineland Adaptive Behavior Scales.

When adjusted for confounders, there was also a negative correlation with socialization subscale on the VABS-II with the RBQ-2 Total score. The RBQ-2 MS was negatively fairly correlated with the socialization subscale score on the VABS-II- this was not seen with the RBQ- RRPRI subscale. Neither the RBQ-2 Total nor its subscales had a significant correlation with the VABS-II motor subscale. These results are represented graphically in Figure 2.

### Relationship between RRBs and social skills

Table 4 reports the correlations between the RBQ-2 and the SRS-2. Significant, positive correlations with fair strength were observed between RBQ-2 Total, RBQ-2 MS and RBQ-2 RRRPI with parent-reported SRS-2 scores (*r* = 0.497, *r* = 0.368, and *r* = 0.431, respectively; all *p* ≤ 0.001). In contrast, there was no meaningful relationship between RBQ-2 Total or its subscales and teacher-reported SRS-2.

**Table 4:** Association between RBQ-2 and SRS-2 scores.

		SRS-2			
		Parent-reported		Teacher-reported	
		*r*	*r*^	*r*	*r^*
RBQ-2	RBQ total	0.496*	0.497**	0.076	0.053
	RBQ-2 MS	0.366*	0.368*	0.027	0.008
	RBQ-2 RRPRI	0.449*	0.431**	0.115	0.079

RBQ-2, Repetitive Behavior Questionnaire, Second Edition; RBQ-MS, RBQ Motor/Sensory Behaviors; RBQ-RRPRI, RBQ Rigidity/Routines/Preoccupation with Restricted Interests; SRS-2, Social Responsiveness Scale, Second Edition. *r*^, adjusted r for gender, age, and positive family history of autism spectrum disorder. *p < 0.05, **p < 0.001. Bolded values are statistically significant results.

## Discussion

Overall, our study results show that in children with ASD, the presence and severity of RRBs are associated with more adverse cognitive and adaptive outcomes (as measured by the MSEL and VABS-II). Even being conservative with our correlation measures, our data is congruent with the known Western literature on this topic (13–17). This is the first Asian study exploring such an association—Asian children with ASD and RRBs appear to experience the same challenges as Caucasian children with this condition.

These previous studies (13, 14, 16) utilized the ADI-R construct as their measure of RRBs—we show the same association in our study using RBQ-2 questionnaire, which is more readily available than the ADI-R. RBQ-2 is an easily available, good and efficient measure of RRBs.

RRBs in autism have been previously categorized into “lower order” and “higher order” behaviors. “Lower order” behaviors describe repetitive motor actions, stereotyped behaviors, physical and/or sensory manipulation of objects and self-injurious behavior, while “higher order” behaviors refer to compulsive ritualistic behaviors, insistence on sameness and circumscribed, fixated interests (33). “Lower order” behaviors have been observed to be more common in younger children and children with language impairment, developmental delay or intellectual disability (17, 34), whereas higher order behaviors involve more advanced cognitive functions (35). Mapped directly to the RBQ-2, “lower order” behaviors are represented by the RBQ-2 MS subscale while “higher order” behaviors are represented by the RBQ-2 RRPRI subscale. Our study describes a group of children with ASD with low cognitive and adaptive functioning as evidenced by lower scores on the MSEL ELC and VABS-II ABC (our scores are 2 standard deviations from the standard mean). In keeping with the findings from the above-mentioned studies, the significant negative correlations of fair strength between the RBQ-2 MS and (i) MSEL ELC, as well as (ii) VABS-II composite score (higher than other correlations between the RBQ scores and other outcomes), suggest a higher frequency of ‘lower order’ RRBs in children with more severe ASD.

Being conservative, of interest, correlations that were of higher strength in our study showed that a higher expression of RRBs, especially those of the “lower order,” was associated with poorer language and communication skills (represented by communication scores on the VABS-II, and the receptive language subscale scores on the MSEL).

This is congruent with impairments in social functioning as seen by the positive correlation of RRBs to parent-reported SRS-2 scores. This is consistent with what we know of in the framework of how RRBs affect cognition and adaptive functioning- by affecting social behavior (19). Although RRBs did not appear to be related to social impairment as rated by teachers (based on the lack of a significant correlation between RBQ-2 and teacher-reported SRS-2 scores), teacher-reported SRS-2 scores were significantly higher than that of parent-reported scores (median of 76.0 and 62.5, respectively; *p* < 0.001). As teachers did not complete the RBQ-2, we were not able to compare RRBs (as reported by teachers) with their report of children’s social functioning in school.

Our study findings also suggest a larger adverse impact of RRBs on language, communication, and social functioning, compared to motor skills development. The relationship between RRBs and social and communication deficits has also been described by Lam et al. (17) where “Repetitive Motor Behaviors” (RMBs) and “Insistence on Sameness” (IS) were associated with greater communication impairments and social deficits (as measured by the Social and Communication subscale scores on the Autism Diagnostic Interview-Revised (ADIR) tool). In our study, we observed that the adaptive skills (represented by the “Daily Living Skills” domain in VABS-II) were impaired.

The association between RBQ-2 Total and poorer motor skills (on both the MSEL and VABS-II) was not statistically significant. However, ‘lower order’ RRBs (represented by the RBQ-MS subscale) did show a significant negative correlation, although this was with weak strength, with the MSEL fine motor subscale. This is consistent with literature where individuals with ASD have been reported to also display motor and coordination abnormalities (36, 37). A functional imaging study in adults with ASD has demonstrated links between RRBs and motor connections in the brain (38); studies on RRBs and their association with motor impairments are otherwise sparse.

On the MSEL, RRBs showed stronger negative correlation with the visual perception subscale compared to language domains. Altered visual perception has been widely reported in individuals with ASD. Feedback connections caused by prior knowledge of true shape can alter visual perception. Smith et al. (39) proposed that the modulatory effect of prior knowledge was different for people with ASD.

In contrast, the lack of statistical significance in the relationships between RBQ-2 RRPRI scores and MSEL and VABS-II scores is likely due to under-representation of children with ASD with less impaired cognitive function in our study population. The over-representation of children with ASD with lower cognitive function in our study may be due to the fact that their parents sought help from our center early due to the severity of their child’s difficulties. In contrast, ASD kids without impaired cognitive function typically present late since they seem to be learning well (40–42). Our study also did not include older children, given that our center sees preschool aged children aged 7 years and below. While this impacts the generalizability of our study findings to all children with ASD, our results describe important clinical associations unique to younger children (aged 7 years and below) with more severe ASD, who should be evaluated and managed differently (vis-à-vis more intensively).

To our knowledge, this is the first Asian study that describes correlations between RRBs and cognitive and adaptive function in young children with ASD. An estimated 1 in 150 children in Singapore are on the autism spectrum (43), similar to the global incidence of 1 in 100 reported by the World Health Organization (44). As this study describes findings in a local Asian population, it can serve as the foundation for further culturally related research and guide clinical practice for management of ASD locally and regionally- particularly given that cultural factors and societal norms can influence the expression and perception of RRBs in children with ASD (23).

Our cross-sectional study describes the correlations between RRBs and adaptive and cognitive function. While the correlation coefficients are not strong, we observed that poorer adaptive skills and cognition in children with ASD were associated with severe RRBs. To investigate if there is a true causative relationship between severity of RRBs and adaptive skills and cognition, randomized interventional studies, with adaptive and cognitive functions as primary outcomes, are necessary.

One limitation of our study is the lack of repeat measures over time. RRBs are known to evolve over time where chronological age is a moderating factor associated with expression and severity of repetitive behaviors (45). Our study population consisted of younger children with ASD [mean age 5.0 (SD 1.2)], where their RRBs are likely to change as they grow older. Evaluating their RRBs at different time points instead of a single time point assessment as in the current study, would provide us with a more comprehensive understanding of the clinical course of these RRBs and their impact over time. Another limitation was that there was a portion of the study sample who did not have complete data for all the study measures as this was an opportunistic clinic-based sample. However, the majority of the sample had data on cognitive and adaptive functioning. We could not perform sensitivity analysis due missing data for some measures. Another limitation was the fact that the results of the study were based solely on the parental report (RBQ-2 questionnaire). Future studies should involve direct observation studies alongside parent questionnaires conducted longitudinally.

Despite our study limitations, our findings add to the literature on RRBs and its potential impact on outcomes in children with ASD, particularly in an Asian setting; they also allude to the need for RRB-specific interventions for children with ASD.

While most ASD-specific early intervention programs focus on social communication (46–48), focused research on intervention practices for repetitive behaviors in ASD is lacking (36). Although not all RRBs may be perceived as negative and debilitating for children with ASDs, they can be challenging to manage (49–51), negatively impact learning and socialization of individuals with ASD (19, 52–55); and, affect family functioning, well-being and quality of life (15, 50, 56). Intervening on RRBs can potentially improve adaptive functioning, increase opportunities for learning and enhance social interaction. As such, there is a growing need for research in the field aimed at addressing the RRB symptom domain in ASD, both internationally and locally.

Boyd et al. (45) have described at least three types of interventions to treat RRBs in ASD, addressing both lower and higher order behaviors. Harrop (11), in her review of evidence-based, parent-mediated interventions for young children with ASD, assessed 29 parent-mediated interventions and their approach to the management, treatment and measurement of restricted and repetitive behaviors. These reviews provide potential behavioral intervention strategies for the management of RRBs in children with ASD. They also support the need for further systemic research in targeting RRBs that can be vastly heterogeneous in their clinical presentation. In another study, Boyd et al. (57) developed Family-Implemented Treatment for Behavior Inflexibility (FITBI), a parent-mediated intervention targeting RRBs over 12 weekly sessions for 1–2 h. They reported significant reductions in repetitive behaviors and increased engagement in more appropriate behaviors. More recently, a randomized clinical trial on managing repetitive behaviors in young children with ASD showed that a parent-mediated, group-based intervention to support parents in recognizing, understanding and managing RRBs in young children with ASD led to a significant reduction in preoccupations with restricted patterns of interest and limited play, and greater parent self-efficacy and improvement in interaction between parent and child (58).

## Conclusion

The presence and severity of RRBs in young children with ASD showed negative correlations with adverse cognitive and adaptive functioning in our study in the domains of language, communication and visual perception. Despite its limitations, this study is the first Asian study and first in Singapore that looked at prevalence of RRBs in local patients with ASD, and their cognitive and adaptive functioning. It also utilized the RBQ-2 questionnaire which is a quick and efficient tool of RRB measure, and is more readily available than the ADOS or ADI-R, thus allowing for easier clinical measure in a busy clinic. We hope that our study will serve as a foundation for further research that would be of interest to both clinicians and researchers - into whether intervening on RRBs through parental education, behavioral and/or pharmacological interventions could reduce the burden of RRBs and potentially improve cognitive outcomes and adaptive skills of children with ASD.

## Data availability statement

The raw data supporting the conclusions of this article will be made available by the authors, without undue reservation.

## Ethics statement

The studies involving humans were approved by National Healthcare Group Domain Specific Review Board [NHG DSRB], Singapore. The studies were conducted in accordance with the local legislation and institutional requirements. Written informed consent for participation in this study was provided by the participants’ legal guardians/next of kin.

## Author contributions

Material preparation, data collection and analysis were performed by WL, YC, MT and KVM. The first draft of the manuscript was written by WL and MT. All authors contributed to the study conception and design, commented on previous versions of the manuscript, read and approved the final manuscript.
